# Approach, avoidance, and affect: a meta-analysis of approach-avoidance tendencies in manual reaction time tasks

**DOI:** 10.3389/fpsyg.2014.00378

**Published:** 2014-05-08

**Authors:** R. Hans Phaf, Sören E. Mohr, Mark Rotteveel, Jelte M. Wicherts

**Affiliations:** ^1^Amsterdam Brain and Cognition Center, University of AmsterdamAmsterdam, Netherlands; ^2^Brain and Cognition Program, Department of Psychology, University of AmsterdamAmsterdam, Netherlands; ^3^Social Psychology Program, Department of Psychology, University of AmsterdamAmsterdam, Netherlands; ^4^Department of Methodology and Statistics, Tilburg UniversityTilburg, Netherlands

**Keywords:** approach, avoidance, affect, arm movement, direct vs. indirect

## Abstract

Approach action tendencies toward positive stimuli and avoidance tendencies from negative stimuli are widely seen to foster survival. Many studies have shown that approach and avoidance arm movements are facilitated by positive and negative affect, respectively. There is considerable debate whether positively and negatively valenced stimuli prime approach and avoidance movements directly (i.e., immediate, unintentional, implicit, automatic, and stimulus-based), or indirectly (i.e., after conscious or non-conscious interpretation of the situation). The direction and size of these effects were often found to depend on the instructions referring to the stimulus object or the self, and on explicit vs. implicit stimulus evaluation. We present a meta-analysis of 29 studies included for their use of strongly positive and negative stimuli, with 81 effect sizes derived solely from the means and standard deviations (combined *N* = 1538), to examine the automaticity of the link between affective information processing and approach and avoidance, and to test whether it depends on instruction, type of approach-avoidance task, and stimulus type. Results show a significant small to medium-sized effect after correction for publication bias. The strongest arguments for an indirect link between affect and approach-avoidance were the absence of evidence for an effect with implicit evaluation, and the opposite directions of the effect with self and object-related interpretations. The link appears to be influenced by conscious or non-conscious intentions to deal with affective stimuli.

## Introduction

Evolutionary reasoning suggests that positive affect acts as a neural code for fitness-enhancing conditions, whereas negative affect acts as a neural code for fitness-reducing conditions (Johnston, 2003; cf. Phaf and Rotteveel, 2012). Tendencies for appetitive and aversive behaviors in response to positive and negative stimuli, respectively, would thus enhance the adaptation of the organism to its environment. Evolutionary computer simulations have indeed shown that approach and avoidance tendencies toward and away from affective stimuli may emerge autonomously in an organism after a number of generations when starting from a completely random organization (for a more detailed description, see den Dulk et al., 2003; Heerebout and Phaf, 2010a,b). In our daily lives, we are often faced with situations that call for quick and appropriate action. Grasping opportunities to obtain a job or avoiding unsafe places at night in big cities are essential behaviors driven by strong emotions. Many emotion theories postulate a fundamental link between emotions and action tendencies, such as, for instance, approach, and avoidance (e.g., Frijda, 1986). Emotions are sometimes assumed to be organized into two different motivational systems that prepare the organism to respond appropriately to emotionally significant stimuli in the environment (Lang et al., 1990). Appetitive motivational circuits are thought to direct the organism to approach positively valenced stimuli, whereas a defensive motivational system would serve to trigger avoidance behavior away from negative stimuli.

In line with this theorizing, a seminal study by Solarz (1960) showed that stimuli with a positive valence foster approach behavior, whereas negative stimuli facilitate avoidance behavior. Participants saw pleasant and unpleasant words on cards that were fixed to a movable stage. They were faster to pull cards with pleasant words toward themselves and to push cards with unpleasant words away from themselves when given feedback what right and wrong responses were. The basic compatibility effect between affect and these action tendencies has later been replicated many times with different types of apparatus, with different reference frames (i.e., self vs. object), with a broad range of affective stimuli, and also with different couplings between push-pull movements and approach-avoidance tendencies (e.g., Chen and Bargh, 1999; De Houwer et al., 2001; Markman and Brendl, 2005; Rinck and Becker, 2007; Saraiva et al., 2013). Duckworth et al. (2002; see also Chen and Bargh, 1999; Alexopoulos and Ric, 2007) even claimed to have found “…definitive evidence that evaluative responding to environmental stimuli can be immediate, unintentional, implicit, and stimulus based. These responses were directly linked to appropriate and adaptive behavioral predispositions toward the stimuli.” (p. 518) In recent years, however, a number of studies has appeared that contested this assumption of complete automaticity (e.g., Rotteveel and Phaf, 2004; Eder et al., 2010). The meta-analysis presented here investigates whether this link between affect and approach-avoidance behavior is direct or indirect (i.e., dependent on instructions, contextual interpretations, or intentions) and whether the divergent results may be due to differences in apparatus, stimuli, experimental design, instructions, and stimulus type.

Many types of emotional stimuli, such as faces and snakes, are not just arbitrary stimuli, but have been relevant to survival during evolutionary history. This may have given them a privileged status in learning processes during ontogeny, which may, for instance, explain why phobias tend to cluster around phylogenetically (e.g., snakes), rather than ontogenetically (e.g., guns), fear-relevant stimuli (Öhman and Mineka, 2001). Such evolutionary prepared, emotional, stimuli may be picked up very quickly and receive processing priority (e.g., Öhman, 1986), influencing subsequent behavior even if they are not perceived fully consciously (e.g., Rotteveel et al., 2001). It should be noted, however, that although there is good evidence that angry faces can be picked up very quickly (e.g., Eastwood et al., 2001), they may be somewhat ambiguous with regard to the action tendencies they evoke. In contrast to a direct link between affect and action tendencies, angry faces seem to require further interpretation to elicit either approach, when evoking anger in the perceiver (e.g., Carver and Harmon-Jones, 2009; Wilkowski and Meier, 2010), or avoidance, when evoking fear (e.g., Rotteveel and Phaf, 2004).

Much affective information processing may indeed proceed automatically. Emotional stimuli can be evaluated automatically, and without conscious processing, on a positive-negative dimension (i.e., affective primacy; Zajonc, 1980). In addition, Chen and Bargh (1999) postulated that automatic affective evaluation automatically predisposes approach and avoidance reactions to affective stimuli. In their second experiment, participants were instructed to pull a lever toward themselves (i.e., approach) or to push it away from themselves (i.e., avoidance) regardless of the stimulus valence. Compatibility effects were found even when participants were not explicitly instructed to evaluate the affective meaning of the presented words. These authors interpreted their finding as demonstrating a direct, automatic link not only between affect and the motivational states of approach and avoidance, but also between motivational states and specific motor actions (i.e., arm flexion and extension). Other studies that had participants evaluate an irrelevant feature of valenced stimuli with a computer joystick (e.g., the background color) seem to support this claim. Many of these studies, however, also provided visual feedback (cf. Seibt et al., 2008). Pulling the joystick increased, and pushing the joystick decreased the size of the stimuli. It can be argued that this zooming feature explicitly reinforces the interpretations of the respective arm movements in terms of approach and avoidance, which puts the complete automaticity of the link between affect, motivational states, and arm movements in question.

The link between affect and approach-avoidance may not be as automatic as Chen and Bargh suggested. Rotteveel and Phaf (2004), for instance, used a vertical stand with three buttons, placed at upper, middle, and lower positions, to measure approach and avoidance behavior. The middle button served as resting (home) button between responses given with the upper or lower buttons. This enabled separate measurement of response initiation times and actual arm movement times (for a similar approach, see Solarz, 1960). Compatibility effects generally only occur in the initiation (i.e., preparation) times but not in the movement times. Pressing the upper button or the lower button corresponds to arm flexion or extension, respectively. In contrast to the explicit evaluation conditions (Experiment 1), no hint of a compatibility effect was found when participants were instructed to evaluate an irrelevant feature (i.e., gender of emotional faces, Experiment 2). The instructions to categorize the gender of the affective faces may not have induced the participants to interpret the flexion and extension movements in affective terms. These experiments differed, however, from the Chen and Bargh studies in other respects, such as type of apparatus and stimulus type (words vs. facial expressions), which may account for the divergent results.

The notion that flexor and extensor movements are associated with approach and avoidance motivations is also supported by a study from Cacioppo et al. (1993) that investigated the influence of flexion and extension on affective evaluation in a reverse direction. The isometric activation of flexor and extensor muscles (i.e., without actually moving the arm) differentially modulated participants' preferences for neutral ideographs (but see Centerbar and Clore, 2006). The authors argued that flexion most often becomes associated with the retrieval or ingestion of something desired, whereas extension is mostly coupled with pushing away something aversive (cf. Maxwell and Davidson, 2007). It remains possible, however, that this link is not automatized or direct, but that the affective interpretation of flexion and extension was set up inadvertently during or in advance of the experiment.

It is not too difficult to conceive of situations where the functions of flexor and extensor movements seem to be reversed. Depending on the particular context, the same physical movement can have different effects resulting in different end-states (e.g., Bamford and Ward, 2008). Nearly touching a spider for instance should elicit the reflex to withdraw the hand (i.e., a flexor movement), particularly for individuals suffering from spider phobia. In addition, approach tendencies can be accompanied by reaching out one's hand, for instance to pet your dog. Seibt et al. (2008) found evidence for this reversal by inducing an object-related frame of reference. Here, a flexor movement represents avoidance of the object, and an extensor movement approach to the object. By instructing participants to move the joystick toward or away from the word on the screen, they obtained opposite compatibility effects. Many studies with object-based instructions have found similar effects that seem to contradict interpretations in terms of a hard-wired relationship between approach-avoidance motivations and particular arm movements (e.g., Markman and Brendl, 2005; Lavender and Hommel, 2007; Eder and Rothermund, 2008). The phrasing of instructions seems to have a large influence on how particular movements of the arm are interpreted by participants. These studies however employ a large range of different apparatus, stimuli, and designs, which may also account for the discrepancies found in literature. In a recent study suggested to us by a reviewer (Saraiva et al., 2013), reference frame (i.e., object vs. self), action tendency (i.e., approach vs. avoidance), and arm/hand movement (push vs. pull) were varied orthogonally in a novel setup. In the self-reference condition participants moved a manikin, presented above or below a central picture, toward or away from it by either pulling or pushing a joystick. In the object-reference condition, the picture was presented either above or below a central manikin. The results largely supported an indirect link by showing that, for the positive pictures at least, self-referent approach was faster than self-referent avoidance and the same was true for object-referent approach and avoidance. Interestingly, for the negative stimuli some muscle specificity remained, but in a direction opposite to the one postulated by Chen and Bargh (1999). When the self-avoided negative pictures, pulling (i.e., flexion) was facilitated relative to pushing (i.e., extension), but extension was faster than flexion, when the self-approached negative pictures.

Evaluative-response-coding accounts (e.g., Eder and Rothermund, 2008) even go so far as to claim that valence has no special status among other stimulus features, such as size, color, and location. Approach and avoidance behaviors are seen to follow general principles of action control, instead of being regulated by distinct motivational mechanisms (Eder and Rothermund, 2008; Lavender and Hommel, 2007). According to this view, compatibility effects are due to a match between evaluative codings of approach and avoidance movements and the affective valence of the stimuli. As discussed above, there is certainly empirical evidence that situational demands influence the meaning of arm movements, which casts doubt on the existence of fixed affective influences on biceps (for arm flexion) and triceps (for arm extension) activations. The commonly used joysticks and levers, however, not only involve muscles in the upper arm, but also pulse and even shoulder muscles, which may be less consistently related to motivational states than specific biceps and triceps activation. Perhaps this type of apparatus leaves more room for interpretation of the context and response coding. In contrast, the vertical button stand (Rotteveel and Phaf, 2004) could potentially be a purer measure of arm flexion and extension. With this apparatus, the instructions to move the under arm vertically, while leaving the upper arm static, are typically not contaminated by references to approach and avoidance (i.e., in the horizontal, sagittal, plane) and flexion and extension do not involve other muscles (e.g., the hand is not turned for pressing the buttons). The vertical stand, moreover, does not move away or toward the self or an object, but holds the distance between the self and the object constant. So, in addition to considering the phrasing of the instructions, comparing different approach-avoidance apparatuses can perhaps shed more light on the main question of this meta-analysis: whether there is a direct or indirect link between affective information processing and approach-avoidance action-tendencies? The following sections describe the moderators included in more detail.

### Task

Apparatuses involving arm and/or hand movements for probing approach-avoidance tendencies may differ in their sensitivity to valence (Krieglmeyer and Deutsch, 2010). Results with the button stand of Rotteveel and Phaf (2004), which was not investigated by Krieglmeyer and Deutsch, may even yield qualitatively different results, due to the movement in the vertical direction instead of in the sagittal direction as with joystick movements. One type of experimental setup only measures approach-avoidance behavior on an abstract level, not involving any physical arm movements (De Houwer et al., 2001). Participants control a manikin on the computer screen that appears randomly above or below a stimulus. By means of manual key presses they either move the manikin toward or away from the stimulus. This setup is referred to here as the abstract-manikin task. Because also more abstract setups are involved in this moderator variable, it will be labeled “task.”

In the joystick task, approach-avoidance behavior is operationalized as horizontally pulling or pushing a vertically positioned control stick. This may involve flexion and extension of the arm, but also pulse and shoulder movements. The same applies to a lever when used as approach-avoidance task (Chen and Bargh, 1999). Because joystick responses suffer from more sideways variability than lever responses, the results from the latter may be slightly more accurate than from the former. The joystick and lever measures are, however, treated as the same task.

The feedback-joystick task, which was discussed above (cf. Seibt et al., 2008), should be considered a separate task. It was shown that due to the visual feedback the task is resistant to cognitive reinterpretations (Rinck and Becker, 2007). When a stimulus-reference point was induced by rephrasing instructions (i.e., pull the joystick away from the picture, push the joystick toward the picture), the compatibility effect did not reverse. Therefore, the crucial aspect in the feedback-joystick task seems to be the visual reinforcement that the stimuli come closer or disappear.

Another distinct task is the vertical three-button stand (Rotteveel and Phaf, 2004). Movements are only made in the vertical direction by either flexing the arm with the biceps muscle or extending the arm with the triceps muscle. Instructions are typically not contaminated with explicit references to approach and avoidance behaviors (i.e., toward and away). Compatibility effects can generally only be observed in the initiation times (the interval between the start of stimulus presentation and resting button release), which reflect the time needed for response preparation (Rotteveel and Phaf, 2004). With respect to the button stand thus only initiation times are involved in this meta-analysis.

The relatively small number of studies that investigated approach-avoidance tendencies toward affective stimuli with whole-body movements (e.g., Stins et al., 2011) did not serve as an extra level to this moderator variable. This would have resulted in a large number of empty cells and deviates somewhat from the lines set out by the seminal Solarz (1960) and Chen and Bargh (1999) studies using arm/hand movements to probe approach and avoidance tendencies. Nevertheless, the Stins et al. results conceptually replicated the results of the latter studies by showing that the initiation of forward steps took more time when evaluating angry than happy faces.

### Instruction

A central question in the literature is whether affective information processing automatically triggers flexion (i.e., approach) and extension (i.e., avoidance), independent from the intention to evaluate the stimuli. Three different instructions were compared to investigate this question. With explicit instructions, as in Solarz (1960) and in Experiment 1 of Chen and Bargh (1999), participants are asked explicitly to evaluate the stimuli with the approach-avoidance task on a positive-negative dimension. They are, for instance, told to pull the joystick toward them or push it away from them when they judge the stimulus as positive or negative, respectively. Implicit instructions do not require participants to attend to stimulus valence. Instead, they are instructed to react to a task-irrelevant feature (e.g., the background color or the gender of a face). The most extreme form of implicitness can be found in research where also the affective valence of the stimuli is implicit (i.e., not consciously recognized by the participant). There are, however, only very few examples of such studies (e.g., Phaf and Rotteveel, 2009; Jones et al., 2011). Nevertheless, “Valence” (explicit vs. implicit) will be included as a separate moderator variable in the meta-analysis. In experimental comparisons explicit instructions tend to yield larger effect sizes than implicit instructions (e.g., Krieglmeyer and Deutsch, 2010).

The third type of instruction will be termed explicit-converted (i.e., relative to the Solarz, 1960, and Chen and Bargh, 1999, studies). Here, the instructions require explicit evaluation, but the meaning of the arm/hand movements is changed, usually by reversing the reference point of flexion and extension (e.g., from the self to the object), or by a relabeling of the end-points of the movements. With object reference flexion of the arm now corresponds to avoidance, whereas extension reflects an approach movement. Other types of instructions are also included in the explicit-converted level, as in Experiment 3 of Eder and Rothermund (2008), where movements to the right or to the left are labeled affectively. Although it would in principle be possible to obtain converted results in implicit evaluation conditions, these conditions have only been tested in combination with explicit evaluation instructions, which means that participants were instructed to respond with the arm-hand movements to the valence of stimuli.

By comparing explicit and explicit-converted conditions, the question can be addressed whether action-tendencies to approach and avoid are context-independent movements consisting of specific motor patterns (i.e., of arm flexion and extension). Furthermore, non-zero effects with implicit instructions would argue for an automatic link between affective information processing and approach-avoidance behavior that does not depend on the intention to evaluate the affective meaning of stimuli.

### Stimuli

In most studies, the same participants were tested with both positively and negatively valenced stimuli. This introduces interdependence between reaction times. In order to extract multiple effect sizes from the same study, positive and negative stimuli were analyzed separately, when possible. Some studies, however, only reported reaction times pooled across compatible (i.e., approach positive and avoid negative stimuli) and incompatible trials (i.e., avoid positive and approach negative stimuli). A third type of analysis was added with the pooled reaction times, where only studies were included that did not report the separate means and standard deviations, or did not provide them upon request. A moderator variable labeled “Design” was also included for comparing within-participants with between-participants manipulations of compatibility.

In the meta-analysis we only included studies using strongly affective stimuli that favored the direct link hypothesis of Chen and Bargh (1999). A primary category of such stimuli may be evolutionary prepared (e.g., emotion faces), which might be more suitable than others (e.g., words) to automatically elicit affect and thus may provide a better opportunity for investigating the automaticity of approach-avoidance tendencies. Emotion words are less likely to be evolutionary prepared, because across languages words denoting the same emotion generally do not share the same perceptual characteristics (e.g., see Phaf and Kan, 2007). Within each analysis (i.e., positive, negative, or both affects), four types of stimuli were investigated. The first type concerned words with an emotional content. These are words that have been selected on the basis of their strong affective valence and thus can be explicitly evaluated on a positive-negative dimension. For this reason studies using individually relevant (e.g., addiction-related, which may be affectively ambiguous; Wiers et al., 2011), and/or weakly valenced stimuli (e.g., social exemplars, Castelli et al., 2004; homophobic words, Clow and Olson, 2010; goal and temptation related words, Fishbach and Shah, 2006) were excluded. In addition, we considered pictures depicting emotional scenes, mostly selected from the International Affective Picture System (IAPS) (Lang et al., 1996). The third type consisted of emotional facial expressions, which are presumably evolutionary prepared stimuli and might therefore be processed more automatically (Öhman, 1986) than for instance words. To ensure comparability across studies, only happy and angry facial expressions were included. The fourth stimulus type involved personally relevant stimuli. Approach-avoidance tasks are often used to assess action-tendencies with stimuli that are relevant to some individual concern. These stimuli were predominantly spider pictures that are tested with participants suffering from a spider phobia.

In sum, we investigated the direct hypothesis in three separate analyses, for positive, negative, and both affects. Krieglmeyer and Deutsch (2010) already provided a direct experimental comparison of some frequently used measures of approach-avoidance, but the field still lacks a quantitative review of the available data. Divergent research results may be caused by confounds of subsidiary factors, such as instruction, apparatus, design, and stimulus, differentially moderating the affective influences on approach-avoidance behaviors. The goal of the current meta-analysis is to provide an estimation of the overall effect size and to shed light on the role of potential moderators. In contrast to an experimental study, however, there is no control in a meta-analysis over the number of replications at a given level of a moderator variable. In addition, the meta-analysis may thus also reveal research areas in need of further study with the approach-avoidance task. Finally, we will investigate whether the literature in this field is subject to publication bias (Rothstein et al., 2005).

## Methods

### Search procedure

A literature search for relevant studies was conducted (until June 2012) across four databases (ISI Web of Science, PsycINFO, PubMed, and Google Scholar) using the search string “(approach-avoidance behavior OR approach-avoidance task OR compatibility) AND evaluation,” with OR and AND representing Boolean operators. The search in PsycINFO resulted in 325 references. In addition, cited reference searches were conducted in ISI Web of Science to search for studies that referred to studies representative for the joystick-lever (Chen and Bargh, 1999, 283 references), the feedback-joystick (Rinck and Becker, 2007, 41 references), and button stand tasks (Rotteveel and Phaf, 2004, 49 references). Additional studies were identified by manual search which consisted of the screening of studies we already knew from our prior research on the approach-avoidance task, and the references therein. Because these provide the best guarantees for study quality, only peer-reviewed, published studies were included in the meta-analysis.

### Inclusion and exclusion criteria

Studies were included according to the following criteria: (1) Studies investigated healthy participants (i.e., not patients). (2) To maximize the chances of finding support for the direct-link hypothesis, only studies with clearly positive and/or negative stimuli were included. (3) Studies involving longer-term moods (e.g., by mood-induction procedures instead of by emotional stimuli) were excluded, but we did include data from control conditions and behavioral assessments prior to a to-be-excluded manipulation (e.g., Roelofs et al., 2005). (4) Studies should employ the joystick/lever, feedback-joystick, abstract tasks, or the button stand as the dependent measure. This resulted in the exclusion of studies that had whole-body movements as the dependent measure (e.g., Stins et al., 2011), or that investigated the reverse effect of arm movements on evaluation of stimuli (e.g., Cacioppo et al., 1993). (5) Studies should report relevant means and standard deviations (or standard errors), which according to Dunlap et al. (1996) should be used rather than *t*-values and other test statistics to compute effect sizes for correlated designs. To prevent a potential inflation of effect sizes, studies that did not report these statistics and from which we could not retrieve the necessary information from the authors were excluded. In a previous meta-analysis (Phaf and Kan, 2007), moreover, we noticed that the discrepancies between effect sizes calculated in the two different manners would sometimes be much larger (in the most extreme case they differed by a factor 10) than suggested by Dunlap and collaborators, possibly due to errors in the statistical analysis. To limit the potential for publication bias due to statistical error, which may be quite prevalent (Bakker and Wicherts, 2011), we therefore had to exclude a number of classical studies that did not report, and where the authors did not provide us with, means and standard deviations (e.g., Duckworth et al., 2002; Seibt et al., 2008; Proctor and Zhang, 2010). Also studies that did not report the results for different levels of a moderator variable separately (e.g., words and pictures, Bamford and Ward, 2008) could not be included in the meta-analysis.

All studies were published between 1999 and mid-2012. Altogether, the meta-analysis included 29 usable studies, from which 81 effect sizes were obtained (Combined *N* = 1538). A detailed overview, listing the studies by moderators, is provided in the supplementary material.

### Effect size calculation

Effect sizes were computed in terms of Cohen's *d* (see Equation 1).

(1) $$d=\frac{M_{inc}-M_{comp}}{S_{pooled}}$$

Cohen's *d* refers to the standardized mean difference between experimental conditions (Hedges and Olkin, 1985; Borenstein et al., 2009), an incompatible condition (*INC*) and a compatible condition (*COMP*), divided by the pooled standard deviation (i.e., Equation 4.4 from Borenstein et al., 2009). Compatible conditions refer to situations where participants approached positive stimuli or avoided negative stimuli, incompatible conditions to situations where participants avoided positive stimuli or approached negative stimuli. In the both affects analysis the reaction times were pooled for the two conditions. With explicit-converted instructions, the coupling between valence and the flexor and extensor movements is usually reversed. In this case, flexor movements to negative stimuli and extensor movements to positive stimuli were considered compatible and the inverse coupling incompatible. This changes the sign with respect to the explicit condition. In the study by Eder and Rothermund (2008; Experiment 3), however, left and right movements were labeled positively and negatively, respectively. These instructions were also coded as explicit-converted instructions in the current meta-analysis, in the sense that this response-label assignment is different from what we refer to as standard explicit instructions.

Cohen's *d* has a slight bias in small samples (Hedges and Olkin, 1985), so we transformed it into Hedges' *g*, using the correction factor *J* (Equation 4.22 from Borenstein et al., 2009). This unbiased estimator, Hedges' *g* (Hedges and Olkin, 1985), was used for subsequent analyses and the correction factor was also applied to sampling variances (Equation 4.24 from Borenstein et al., 2009). Because the majority of studies had repeated measures designs (*k* = 26), the variance of *g* was computed with Equation 4.28 from Borenstein et al. (2009), which requires the correlation (*r*) between pairs of observations (see Dunlap et al., 1996). For those studies using an independent groups (i.e., between-participants) design, the sampling variance of *g* was computed with Equation 4.20 from Borenstein et al. (2009). All studies in the current meta-analysis with independent groups had equal sample sizes per group.

### Missing data

Whenever necessary, authors were contacted to gather means and standard deviations in order to compute effect sizes. It is not common practice to also report *r*, the correlation between pairs of observations in repeated-measures designs. Therefore, *r* was estimated from paired *t*-tests according to Equation 2.

(2) $$r=\frac{2t^{2}-g^{2}n}{2t^{2}}$$

It could, similarly, be calculated from repeated measures ANOVAs according to Equation 3.

(3) $$r=\frac{2F-g^{2}n}{2F}$$

For some studies (Phaf and Rotteveel, 2009; Seidel et al., 2010a,b), *r* could be computed from the raw data and compared to estimations derived from the test-statistics. The estimations turned out to be fairly accurate, validating the use of the above formulas. For the remaining studies that did not report the relevant test-statistics the average of all available correlations, weighted by individual sample sizes, was imputed as the correlation for that individual study. If means and standard deviations were not provided, and if the correlation between measures was not reported, nor could be estimated appropriately, the study was excluded as recommended by Dunlap et al. (1996).

### Data analysis

Analyses were performed in the statistical software package R (version 2.14.1) (R Development Core Team, 2010) with the metafor package (Viechtbauer, 2010). Due to expected heterogeneity, all analyses were computed within the random-effects model. The proportion of systematic unexplained variance (τ^2^) was estimated using restricted maximum-likelihood estimation, which is approximately unbiased and quite efficient (Viechtbauer, 2010). Cochran's *Q*-test (Hedges and Olkin, 1985) served to test the null hypothesis of homogeneity of the effect sizes. A significant Cochran's *Q*-test indicated study heterogeneity. Influence analysis (i.e., the exclusion of single studies) was performed to identify influential studies based on Cook's distance and residual heterogeneity.

Separate analyses were performed for positive affect, negative affect, and both affects. Moderating variables were defined a priori. Hypothesized categorical moderators were (1) *task:* vertical button stand, joystick/lever, feedback-joystick, abstract-manikin task; (2) *instruction:* explicit (i.e., task-relevant), explicit-converted (i.e., task-relevant), implicit (i.e., task-irrelevant); (3) *stimulus type:* emotional facial expressions, emotional words, emotional pictures, personally relevant stimuli: (4) *design:* repeated measures design, independent groups design; (5) *valence:* explicitly valenced stimuli, implicitly valenced stimuli. The statistic *Q*_*m*_ served as an omnibus test for differences between levels, *Q*_*e*_ as a test for residual heterogeneity.

### Publication bias

An important issue in meta-analyses is the occurrence of a publication bias (Rothstein et al., 2005). Studies with statistically significant effects and positive treatment outcomes are more likely to be published than null results. If a publication bias is present, the studies included in the meta-analysis are not representative of all valid studies undertaken in the field, leading to an over-estimation of the effect. If studies with non-significant results remain unpublished, this may be reflected in an asymmetric funnel plot and an excess of significant findings (Sterne and Egger, 2005; Ioannidis and Trikalinos, 2007; Bakker et al., 2012; Francis, 2012). In the graph a measure of the accuracy of the study is plotted against the effect size. In the absence of publication bias, studies should be scattered symmetrically around the most accurate studies in a pyramid fashion. In the present meta-analysis, the occurrence of publication bias was tested by conducting a regression test for funnel plot asymmetry within relatively homogenous subsets of studies (Egger et al., 1997). To correct for a possible publication bias, the trim-and-fill method was applied to the same subsets (Duval and Tweedie, 2000). This method estimates the number of missing studies and provides an adjustment of the overall effect size.

## Results

### Positive affect

The effect sizes (*k* = 27) ranged from *g* = −0.08 to 1.29. The random effects model yielded a significant average effect size (*g* = 0.307; *p* < 0.0001; 95% CI = 0.200, 0.414). The majority of the effect sizes were in the expected direction (*k* = 25). Twelve of these positive effect sizes were significant. Two studies showed an effect in the opposite direction, one of which was significant. The estimated amount of heterogeneity was equal to τ^2^ = 0.057; 95% CI = 0.029, 0.148. There was a clear indication of heterogeneity in effect sizes (*Q* = 183.24, *df* = 26, *p* < 0.0001). Influence analysis identified three outliers, *g* = 1.29 (*standardized* residual = 0.983; Markman and Brendl, 2005; A), *g* = 1.06 (*standardized* residual = 0.755; Markman and Brendl, 2005; B), *g* = 0.8 (*standardized* residual = 0.501; Phaf and Rotteveel, 2009; Experiment 2A). The exclusion of the three outliers reduced the average effect size (*g* = 0.216, *p* < 0.0001; 95% CI = 0.141, 0.292). The unexplained variance component was reduced (τ^2^= 0.0172), but the test of heterogeneity was still significant (*Q* = 93.03, *df* = 23, *p* < 0.0001). All moderator analyses were conducted under exclusion of the three outliers, except for the analysis of the moderator *valence*, because one outlier (Phaf and Rotteveel, 2009; A) was the only study using stimuli with implicit valence.

### Moderator analyses of positive affect

Four moderator variables (*task, instruction, stimulus type, design*) were included in a mixed effects model. The estimated amount of residual heterogeneity was equal to τ^2^ = 0.0000; 95% CI = 0.0000, 0.0044, suggesting that at least 74% of the variance in effect sizes could be accounted for by including the moderators (*Q*_*m*_ = 83.4099, *df* = 7, *p* < 0.0001). The test for residual heterogeneity was not significant (*Q*_*e*_ = 9.6185, *df* = 16, *p* = 0.8858).

The results of the moderator analyses are provided in Table 1. The abstract-manikin task did not occur in the studies investigating positive affect separately. The test of the moderator *task* was significant (*Q*_*m*_ = 6.54, *df* = 2, *p* = 0.038). The average effect size was significantly different from zero for the vertical stand (*g* = 0.272; *p* < 0.001; 95% CI = 0.113, 0.432), as well as for the joystick/lever (*g* = 0.251; *p* < 0.0001; 95% CI = 0.165, 0.337). The feedback-joystick did not yield a significant effect (*g* = 0.047; *p* = 0.519; 95% CI = −0.095, 0.189), which is probably a consequence of many studies using the feedback-joystick in combination with implicit instructions. There was a significant difference between the feedback-joystick and the vertical stand (*p* = 0.039), and between the feedback-joystick and the joystick/lever (*p* = 0.016). The moderator *task* explained 35% of the variance (τ^2^ = 0.011). The test for residual heterogeneity was significant (*Q*_*e*_ = 38.70, *df* = 21, *p* = 0.011).

**Table 1:** **Results of moderator analyses of positive affect**.

**Moderator**	**Level**	***k***	**Estimate**	**[95% CI]**	***p***	***p* for diff**
Task	Feedback (Ref)	4	0.047	[−0.095, 0.189]	0.519	
	Stand	6	0.272	[0.113, 0.432]	0.0008	0.039
	Stick	14	0.251	[0.165, 0.337]	<0.0001	0.016
Instruction	Implicit (Ref)	7	0.028	[−0.069, 0.126]	0.572	
	Explicit	14	0.287	[0.204, 0.369]	<0.0001	<0.0001
	Explicit-converted	3	0.287	[0.146, 0.429]	<0.0001	0.0031
Stimulus type	Faces (Ref)	15	0.148	[0.056, 0.241]	0.0017	
	Pictures	4	0.203	[0.022, 0.383]	0.028	0.600
	Words	5	0.339	[0.211, 0.467]	<0.0001	0.018
Design	Independent	1	0.677	[−0.107, 1.460]	0.091	
	Repeated	23	0.212	[0.137, 0.287]	<0.0001	0.247
Valence	Explicit	24	0.216	[0.141, 0.292]	<0.0001	
	Implicit	1	0.808	[0.486, 1.130]	<0.0001	0.0005

*Note. Ref, reference level that was deemed most informative for the comparison; k, number of studies; [95% CI], 95% confidence interval; p, p-value for each level; p for diff, p-value for difference between respective level and reference level. Task: Stand, vertical button stand; Joystick, joystick/lever; Feedback, feedback-joystick. Stimulus type: Words, emotional words; Pictures, emotional pictures; Faces, emotional facial expressions. Design: Repeated, repeated measures design; Independent, independent groups design. Valence: Explicit, explicitly valenced stimuli; Implicit, implicitly valenced stimuli*.

The test of the moderator *instruction* was significant (*Q*_*m*_ = 17.62, *df* = 2, *p* = 0.0001). The average effect size differed significantly from zero for explicit instructions (*g* = 0.287; *p* < 0.0001; 95% CI = 0.204, 0.369) and for explicit-converted instructions (*g* = 0.287; *p* < 0.0001; 95% CI = 0.146, 0.429), but not for implicit instructions (*g* = 0.028; *p* = 0.572). The average effect size was significantly smaller for implicit instructions than for explicit instructions (*p* < 0.0001) and for explicit-converted instructions (*p* = 0.003). The moderator *instruction* explained 66% of the variance. The test for residual heterogeneity was not significant (*Q*_*e*_ = 27.75, *df* = 21, *p* = 0.147).

Considering the levels moderator *stimulus type* separately, the average effect size was significant for emotional words (*g* = 0.339; *p* < 0.0001; 95% CI = 0.211, 0.467), as well as for emotional pictures (*g* = 0.203; *p* = 0.028; 95% CI = 0.022, 0.383), and for emotional facial expressions (*g* = 0.148; *p* = 0.002; 95% CI = 0.056, 0.241). Only the difference between emotional words and facial expressions was significant (*p* = 0.018), which might also be due to many studies using facial expressions in combination with implicit instructions. However, the omnibus test of the moderator was not significant (*Q*_*m*_ = 5.62, *df* = 2, *p* = 0.060).

The test of the moderator *design* was not significant (*Q*_*m*_ = 1.34, *df* = 1, *p* = 0.247), presumably due to the low number of studies having an independent groups design. The test of the moderator *valence* was significant (*Q*_*m*_ = 12.27, *df* = 1, *p* < 0.001). Only one study used implicitly affective stimulus material (i.e., arrows) and showed a significantly larger effect (*g* = 0.808; *p* < 0.0001; 95% CI = 0.486, 1.130) than all other studies (*g* = 0.216; *p* < 0.0001; 95% CI = 0.141, 0.292). The moderator *valence* explained 45% of the variance. The test for residual heterogeneity was significant (*Q*_*e*_ = 93.03, *df* = 23, *p* < 0.0001).

### Subset analyses of positive affect

Because the above moderator analyses indicated no significant effect for implicit instructions, some effect sizes may have been underestimated, when relatively many studies at this moderator level had such instructions. It therefore seems worthwhile to examine interactions between moderators. For this purpose, however, there needs to be at least one observation for every combination of moderator levels, which was not the case in this dataset, particularly for the implicit instructions. Therefore, we performed analyses under exclusion of all seven studies using implicit instructions.

After exclusion of studies with implicit instructions, the random effects model yielded a significant average effect size (*g* = 0.283; *p* < 0.0001; 95% CI = 0.216, 0.348). The estimated amount of heterogeneity equaled τ^2^ = 0.0037; 95% CI = 0, 0.0211. The test for heterogeneity was not significant (*Q* = 17.74, *df* = 16, *p* = 0.340), confirming that most of the heterogeneity was due to differences in average effect sizes between task-irrelevant instructions (implicit) and task-relevant instructions (explicit and explicit-converted).

As expected, moderator analyses for the subset of task-relevant instructions (see Table 2) showed that no moderator was significant (except for the moderator stimulus *valence*). This suggests that differences in effect size between levels of the moderators appeared due to the inclusion of implicit instructions. Specifically, the average effect size was now significant for the feedback-joystick (*g* = 0.233, *p* = 0.035, 95% CI = 0.016, 0.451). The average effect size was not significantly different from the joystick/lever (*p* = 0.690) and the vertical stand (*p* = 0.463). The same applies to the moderator *stimulus type*. There was no statistically significant difference in effect size between emotional facial expressions and emotional words (*p* = 0.707).

**Table 2:** **Results of subset analyses of positive affect after exclusion of studies with implicit instructions**.

**Moderator**	**Level**	***k***	**Estimate**	**[95% CI]**	***P***	***p* for diff**
Task	Feedback (Ref)	1	0.233	[0.016, 0.451]	0.035	
	Stand	5	0.337	[0.168, 0.505]	<0.0001	0.463
	Stick	11	0.281	[0.200, 0.362]	<0.0001	0.690
Instruction	Explicit	14	0.286	[0.205, 0.368]	<0.0001	
	Explicit-converted	3	0.284	[0.147, 0.422]	<0.0001	0.981
Stimulus type	Faces (Ref)	8	0.290	[0.164, 0.417]	<0.0001	
	Pictures	4	0.199	[0.045, 0.353]	0.011	0.369
	Words	5	0.322	[0.220, 0.423]	<0.0001	0.707
Design	Independent	1	0.677	[−0.073, 1.426]	0.077	
	Repeated	16	0.279	[0.214, 0.345]	<0.0001	0.301
Valence	Explicit	17	0.283	[0.217, 0.348]	<0.0001	
	Implicit	1	0.808	[0.580, 1.036]	<0.0001	<0.0001

*Note. Ref, reference level that was deemed most informative for the comparison; k, number of studies; [95% CI], 95% confidence interval; p, p-value for each level; p for diff, p-value for difference between respective level and reference level. Task: Stand, vertical button stand; Joystick, joystick/lever; Feedback, feedback-joystick. Stimulus type: Words, emotional words; Pictures, emotional pictures; Faces, emotional facial expressions. Design: Repeated, repeated measures design; Independent, independent groups design. Valence: Explicit, explicitly valenced stimuli; Implicit, implicitly valenced stimuli*.

### Negative affect

Effect sizes (*k* = 32) ranged from *g* = −0.13 to 1.85. Four studies showed an effect in a direction opposite to the expected one. However, none of these effect sizes was significant. The remaining effect sizes were in the expected direction (*k* = 28). Similar to the analysis of positive affect, the random effects model yielded a significant average effect size (*g* = 0.304; *p* < 0.0001; 95% CI = 0.174, 0.435). The estimated amount of heterogeneity was equal to τ^2^ = 0.122; 95% CI = 0.082, 0.306. There was a clear indication of heterogeneity in effect sizes (*Q* = 189.97, *df* = 31, *p* < 0.0001). Influence analysis identified two outliers, *g* = 1.85 (*standardized* residual = 1.543; Markman and Brendl, 2005; D), *g* = 1.76 (*standardized residual* = 1.457; Markman and Brendl, 2005; C). The exclusion of the two outliers resulted in a reduced average effect size (*g* = 0.217; *p* < 0.0001; 95% CI = 0.141, 0.292). The unexplained variance component was reduced (τ^2^ = 0.029), but the test for heterogeneity was still significant (*Q* = 104.32, *df* = 29, *p* < 0.0001). Moderator analyses were conducted under exclusion of the two outliers.

### Moderator analyses of negative affect

All five moderator variables (*task, instruction, stimulus type, design, valence*) were included in a mixed effects model. The abstract-manikin level again did not occur in the studies that investigated negative affect separately. The results of the moderator analyses are provided in Table 3. The estimated amount of residual heterogeneity was equal to τ^2^ = 0.0234. However, the test of the moderators was not significant (*Q*_*m*_ = 13.7224, *df* = 9, *p* = 0.1325). There was substantial residual heterogeneity (*Q*_*e*_ = 56.8393, *df* = 20, *p* < 0.0001). Separate analyses of each moderator confirmed that no moderator was significant, which means that none of the levels differed significantly from the other levels of the same moderator.

**Table 3:** **Results of moderator analyses of negative affect**.

**Moderator**	**Level**	***k***	**Estimate**	**[95% CI]**	***p***	***p* for diff**
Task	Feedback (Ref)	8	0.212	[0.057, 0.367]	0.0074	
	Stand	7	0.184	[0.021, 0.347]	0.0269	0.810
	Stick	15	0.235	[0.126, 0.344]	<0.0001	0.810
Instruction	Implicit (Ref)	10	0.103	[−0.018, 0.225]	0.0959	
	Explicit	17	0.249	[0.159, 0.339]	<0.0001	0.059
	Explicit-converted	3	0.389	[0.155, 0.624]	0.0012	0.034
Stimulus type	Faces (Ref)	16	0.134	[0.034, 0.235]	0.0089	
	Pictures	5	0.322	[0.143, 0.502]	0.0279	0.074
	Words	5	0.277	[0.119, 0.434]	0.0006	0.136
	Relevant	4	0.348	[0.107, 0.589]	0.0047	0.110
Design	Independent	1	0.504	[−0.301, 1.308]	0.220	
	Repeated	29	0.214	[0.138, 0.290]	<0.0001	0.482
Valence	Explicit	24	0.203	[0.130, 0.2761]	<0.0001	
	Implicit	1	0.518	[0.159, 0.8775]	0.0047	0.092

*Note. Ref, reference level that was deemed most informative for the comparison; k, number of studies; [95% CI], 95% confidence interval; p, p-value for each level; p for diff, p-value for difference between respective level and reference level. Task: Stand, vertical button stand; Joystick, joystick/lever; Feedback, feedback-joystick. Stimulus type: Words, emotional words; Pictures, emotional pictures; Faces, emotional facial expressions; Relevant, personally relevant stimuli. Design: Repeated, repeated measures design; Independent, independent groups design. Valence: Explicit, explicitly valenced stimuli; Implicit, implicitly valenced stimuli*.

The test of the moderator *task* was not significant (*Q*_*m*_ = 0.26, *df* = 2, *p* = 0.876). The average effect size differed significantly from zero for the joystick/lever (*g* = 0.235; *p* < 0.0001; 95% CI = 0.126, 0.344), for the feedback-joystick (*g* = 0.212; *p* = 0.007; 95% CI = 0.057, 0.367), and for the vertical stand (*g* = 0.184; *p* = 0.027; 95% CI = 0.021, 0.347).

The omnibus test for moderation due to different instructions was close to significance (*Q*_*m*_ = 5.91, *df* = 2, *p* = 0.052). Effect sizes differed significantly from zero for explicit-converted instructions (*g* = 0.389; *p* = 0.001; 95% CI = 0.155, 0.624) and for explicit instructions (*g* = 0.249; *p* < 0.0001; 95% CI = 0.159, 0.339). Implicit instructions did not yield a significant effect (*g* = 0.103; *p* = 0.0959). Results indicated a significant difference between implicit and explicit-converted instructions (*p* = 0.034) and there was a trend toward a significant difference between implicit and explicit instructions (*p* = 0.059). There was no difference between explicit and explicit-converted instructions (*p* = 0.276). This pattern is consistent with the results of the analysis of positive affect. In order to increase power, the two levels of explicit and explicit-converted instructions were combined to form the level task-relevant instructions. The test of this moderator was significant (*Q*_*m*_ = 4.74, *df* = 1, *p* = 0.030). Task-relevant instructions showed a significant average effect size (*g* = 0.267; *p* < 0.0001; 95% CI = 0.183, 0.351), whereas task-irrelevant (implicit) instructions did not (*g* = 0.103; *p* = 0.096; 95% CI = −0.018, 0.225).

The omnibus test of the moderator *stimulus type* was not significant (*Q*_*m*_ = 5.58, *df* = 3, *p* = 0.134). The effect size was significantly different from zero for personally relevant stimuli (*g* = 0.348; *p* = 0.005; 95% CI = 0.107, 0.589), for emotional pictures (*g* = 0.322; *p* < 0.001; 95% CI = 0.143, 0.502), for emotional words (*g* = 0.277; *p* < 0.001; 95% CI = 0.119, 0.434), and for emotional facial expressions (*g* = 0.134; *p* = 0.009; 95% CI = 0.034, 0.235). The test of the moderator *design* was not significant (*Q*_*m*_ = 0.49, *df* = 1, *p* = 0.4824). One study used implicitly affective stimulus material (i.e., arrows; Phaf and Rotteveel, 2009). The effect size for this study was not significantly different from the effect size of all other studies (*Q*_*m*_ = 2.84, *df* = 1, *p* = 0.092).

### Subset analyses of negative affect

After recoding the moderator *instruction*, results showed a significant difference between task-relevant and implicit instructions. Although all other moderators were not significant, the magnitude of the average effect size for each level might depend on which instructions were used. Therefore, subset analyses were performed under exclusion of all studies employing implicit instructions.

After exclusion of 10 studies with implicit instructions the random effects model yielded a significant average effect size (*g* = 0.265; *p* < 0.0001; 95% CI = 0.188, 0.343). The estimated amount of heterogeneity was equal to τ^2^ = 0.018; 95% CI = 0.006, 0.062. The test for heterogeneity in effect sizes was significant (*Q* = 60.92, *df* = 19, *p* < 0.0001).

Excluding studies with implicit instructions did not affect the significance of any of the omnibus moderator tests. The magnitude of some average effect sizes was increased, however, due to the exclusion of implicit instructions (see Table 4). Differences between levels of the moderator *task* were numerically reduced. One study using personally relevant stimuli, moreover showed a considerably larger effect size (*g* = 0.769, *p* = 0.006, 95% CI = 0.222, 1.316).

**Table 4:** **Results of subset analyses of negative affect after exclusion of studies with implicit instructions**.

**Moderator**	**Level**	***k***	**Estimate**	**[95% CI]**	***p***	***p* for diff**
Task	Feedback (Ref)	2	0.244	[−0.017, 0.505]	0.067	
	Stand	6	0.251	[0.094, 0.408]	0.0017	0.964
	Stick	12	0.278	[0.173, 0.382]	<0.0001	0.815
Instruction	Explicit	17	0.248	[0.165, 0.331]	<0.0001	
	Explicit-converted	3	0.385	[0.169, 0.601]	0.0005	0.246
Stimulus type	Faces (Ref)	9	0.202	[0.076, 0.328]	0.0017	
	Pictures	5	0.317	[0.158, 0.476]	<0.0001	0.266
	Words	5	0.272	[0.135, 0.408]	<0.0001	0.463
	Relevant	1	0.769	[0.222, 1.316]	0.0058	0.048
Design	Independent	1	0.504	[−0.273, 1.281]	0.204	
	Repeated	19	0.263	[0.185, 0.341]	<0.0001	0.546
Valence	Explicit	19	0.247	[0.173, 0.320]	<0.0001	
	Implicit	1	0.518	[0.227, 0.809]	0.0005	0.076

*Note. Ref, reference level that was deemed most informative for the comparison; k, number of studies; [95% CI], 95% confidence interval; p, p-value for each level; p for diff, p-value for difference between respective level and reference level. Task: Stand, vertical button stand; Joystick, joystick/lever; Feedback, feedback-joystick. Stimulus type: Words, emotional words; Pictures, emotional pictures; Faces, emotional facial expressions; Relevant, personally relevant stimuli. Design: Repeated, repeated measures design; Independent, independent groups design. Valence: Explicit, explicitly valenced stimuli; Implicit, implicitly valenced stimuli*.

### Sensitivity analyses

In order to investigate the impact of the correlation between pairs of observations, sampling variances were computed based on the two most extreme correlations. This was done separately for each analysis. For the analysis of positive affect, the lowest correlation derived from test-statistics was equal to *r* = 0.479 (Seidel et al., 2010b; A). The highest correlation was equal to *r* = 0.797 (Van Dantzig et al., 2008; A). The results from the random effects model with the lowest correlation (*g* = 0.310; 95% CI = 0.201, 0.420; *Q* = 181.17; τ^2^ = 0.0584) were very similar to those with the highest correlation (*g* = 0.299; 95% CI = 0.199, 0.400; *Q* = 192.65; τ^2^ = 0.0546).

For the analysis of negative affect, the lowest correlation derived from test-statistics was equal to *r* = 0.403 (Rinck and Becker, 2007; Study 1). The highest correlation was equal to *r* = 0.966 (Van Dantzig et al., 2008; B). The results from the random effects model with the lowest correlation (*g* = 0.313; 95% CI = 0.172, 0.453; *Q* = 166.76; τ^2^ = 0.1297) were very similar to those with the highest correlation (*g* = 0.300; 95% CI = 0.170, 0.430; *Q* = 440.74; τ^2^ = 0.1267). In sum, these sensitivity analyses suggest that the estimated mean effect sizes are hardly affected by the use of alternative imputations of the correlations between pairs of observations in within-participants designs. All studies in the both affects analysis reported the relevant test statistic, so that the correlations and effect sizes could be estimated here with a relatively large precision.

### Both affects

The effect sizes (*k* = 22) ranged from *g* = 0.002 to 0.87. All effect sizes were in the expected direction. Eighteen of these effect sizes were significant. Influence analysis identified no outliers. The random effects model yielded a significant average effect size (*g* = 0.308; *p* < 0.0001; 95% CI = 0.205, 0.410). The estimated amount of heterogeneity was equal to τ^2^ = 0.045; 95% CI = 0.0213, 0.1107. The test for heterogeneity was significant (*Q* = 133.13, *df* = 21, *p* < 0.0001).

### Moderator analyses of both affects

The same five moderators were analyzed in a mixed effects model. The results of the analyses are provided in Table 5. The estimated amount of residual heterogeneity was equal to τ^2^= 0.0007; 95% CI = 0, 0.0436, suggesting that 98.4% of the systematic variance in effect sizes could be accounted for by including the moderators (*Q*_*m*_ = 104.4251, *df* = 9, *p* < 0.0001). The test for residual heterogeneity was not significant (*Q*_*e*_ = 15.8157, *df* = 12, *p* = 0.1998).

**Table 5:** **Results of moderator analyses of both affects**.

**Moderator**	**Level**	***k***	**Estimate**	**[95% CI]**	***p***	***p* for diff**
Task	Feedback (Ref)	1	0.094	[−0.323, 0.511]	0.659	
	Abstract	3	0.281	[0.030, 0.532]	0.028	0.450
	Stand	1	0.662	[0.212, 1.111]	0.0039	0.069
	Stick	17	0.305	[0.187, 0.423]	<0.0001	0.340
Instruction	Implicit (Ref)	6	0.076	[−0.027, 0.180]	0.1447	
	Explicit	9	0.403	[0.286, 0.521]	<0.0001	<0.0001
	Explicit-converted	7	0.433	[0.295, 0.571]	<0.0001	<0.0001
Stimulus type	Faces (Ref)	3	0.146	[−0.123, 0.415]	0.2861	
	Pictures	4	0.321	[0.071, 0.571]	0.0119	0.352
	Words	15	0.343	[0.215, 0.472]	<0.0001	0.194
Design	Independent	2	0.749	[0.190, 1.305]	0.0086	
	Repeated	20	0.291	[0.190, 0.393]	<0.0001	0.115
Valence	Explicit	20	0.297	[0.187, 0.406]	<0.0001	
	Implicit	2	0.409	[0.088, 0.730]	0.0126	0.517

*Note. Ref, reference level that was deemed most informative for the comparison; k, number of studies; [95% CI], 95% confidence interval; p, p-value for each level; p for diff, p-value for difference between respective level and reference level. Task: Stand, vertical button stand; Joystick, joystick/lever; Feedback, feedback-joystick; Abstract, abstract manikin task. Stimulus type: Words, emotional words; Pictures, emotional pictures; Faces, emotional facial expressions. Design: Repeated, repeated measures design; Independent, independent groups design. Valence: Explicit, explicitly valenced stimuli; Implicit, implicitly valenced stimuli*.

The test of the moderator *task* was not significant (*Q*_*m*_ = 3.44, *df* = 3, *p* = 0.328). The average effect size differed significantly from zero for the vertical stand (*g* = 0.662; *p* = 0.004; 95% CI = 0.212, 1.11), the joystick/lever (*g* = 0.305; *p* < 0.0001; 95% CI = 0.187, 0.423), and the abstract-manikin task (*g* = 0.281; *p* = 0.028; 95% CI = 0.030, 0.532). The feedback-joystick task did not yield a significant effect (*g* = 0.094; *p* = 0.659).

The test of the moderator *instruction* was significant (*Q*_*m*_ = 23.71, *df* = 2, *p* < 0.0001). The average effect size differed significantly from zero for explicit-converted instructions (*g* = 0.433; *p* < 0.0001; 95% CI = 0.295, 0.571) and explicit instructions (*g* = 0.403; *p* < 0.0001; 95% CI = 0.286, 0.521). Their difference was not significant (*p* = 0.747). Implicit instructions did not yield a significant effect (*g* = 0.076; *p* = 0.148). The average effect size was significantly smaller with implicit instructions than with explicit instructions (*p* < 0.0001) and explicit-converted instructions (*p* < 0.0001). The moderator *instruction* explained 67% of the variance (τ^2^ = 0.0150). The test for residual heterogeneity was significant (*Q*_*e*_ = 50.56, *df* = 21, *p* = 0.0001).

The test of the moderator *stimulus type* was not significant (*Q*_*m*_ = 1.70, *df* = 2, *p* = 0.429). The average effect size differed significantly from zero for emotional words (*g* = 0.343; *p* < 0.0001; 95% CI = 0.215, 0.472), and for emotional pictures (*g* = 0.321; *p* = 0.012; 95% CI = 0.071, 0.571), but not for emotional facial expressions (*g* = 0.146; *p* = 0.286). The test of the moderator *design* was not significant (*Q*_*m*_ = 2.49, *df* = 1, *p* = 0.115). Two studies employed implicitly affective stimulus material. The average effect size for these studies was not significantly different from the average effect size of all other studies (*Q*_*m*_ = 0.420, *df* = 1, *p* = 0.517).

### Subset analyses of both affects

The moderator analyses so far have demonstrated consistently that task-relevant (explicit or explicit-converted) instructions are required to find an effect of affective information processing on approach-avoidance behaviors. Accordingly, the analysis of both affects also showed a non-significant effect for task-irrelevant (implicit) instructions. Again, subset analyses were performed under exclusion of all studies using implicit instructions in order to investigate how they might affect the results (see Table 6). However, inferences are based on even fewer studies and should therefore be treated with caution. After exclusion of the six studies with implicit instructions the random effects model yielded a significant medium average effect size (*g* = 0.425; *p* < 0.0001; 95% CI = 0.317, 0.533). The estimated amount of heterogeneity was equal to τ^2^ = 0.0283; 95% CI = 0.007, 0.088. The test for heterogeneity in effect sizes was significant (*Q* = 44.98, *df* = 15, *p* < 0.0001).

**Table 6:** **Results of subset analyses of both affects**.

**Moderator**	**Level**	***k***	**Estimate**	**[95% CI]**	***p***	***p* for diff**
Task	Abstract (Ref)	1	0.729	[0.373, 1.084]	<0.0001	
	Stand	1	0.662	[0.348, 0.975]	<0.0001	0.782
	Stick	14	0.368	[0.268, 0.468]	<0.0001	0.056
Instruction	Explicit	9	0.415	[0.267, 0.562]	<0.0001	
	Explicit-converted	7	0.443	[0.271, 0.614]	<0.0001	0.809
Stimulus type	Faces (Ref)	2	0.210	[−0.067, 0.487]	0.1376	
	Pictures	3	0.423	[0.176, 0.670]	0.0008	0.260
	Words	11	0.474	[0.342, 0.606]	<0.0001	0.091
Design	Independent	2	0.749	[0.219, 1.280]	0.0056	
	Repeated	14	0.411	[0.302, 0.520]	<0.0001	0.221
Valence	Explicit	15	0.431	[0.308, 0.555]	<0.0001	
	Implicit	2	0.406	[0.130, 0.682]	0.0039	0.872

*Note. Ref, reference level that was deemed most informative for the comparison; k, number of studies; [95% CI], 95% confidence interval; p, p-value for each level; p for diff, p-value for difference between respective level and reference level. Task: Stand, vertical button stand; Joystick, joystick/lever; Abstract, abstract manikin task. Stimulus type: Words, emotional words; Pictures, emotional pictures; Faces, emotional facial expressions. Design: Repeated, repeated measures design; Independent, independent groups design. Valence: Explicit, explicitly valenced stimuli; Implicit, implicitly valenced stimuli*.

The test of the moderator *task* now reached significance (*Q*_*m*_ = 6.22, *df* = 2, *p* = 0.045). The level feedback-joystick was dropped, because the only study in this level used implicit instructions. There was only one study that used the abstract-manikin task (*g* = 0.729, *p* < 0.0001, 95% CI = 0.373, 1.084). This effect size was almost significantly larger (*p* = 0.056) than for the joystick/lever (*g* = 0.368, *p* < 0.0001, 95% CI = 0.2681, 0.468). The moderator *task* explained 42% of the variance (τ^2^ = 0.0164). The test for residual heterogeneity was significant (*Q*_*e*_ = 27.00, *df* = 13, *p* = 0.0124). The test of the moderator *stimulus type* was not significant (*Q*_*m*_ = 2.85, *df* = 2, *p* = 0.240). Based on two studies, the average effect size for emotional facial expressions was still not significant (*g* = 0.210, *p* = 0.138, 95% CI = 0.067, 0.487).

### Publication bias

**Figure 1:**
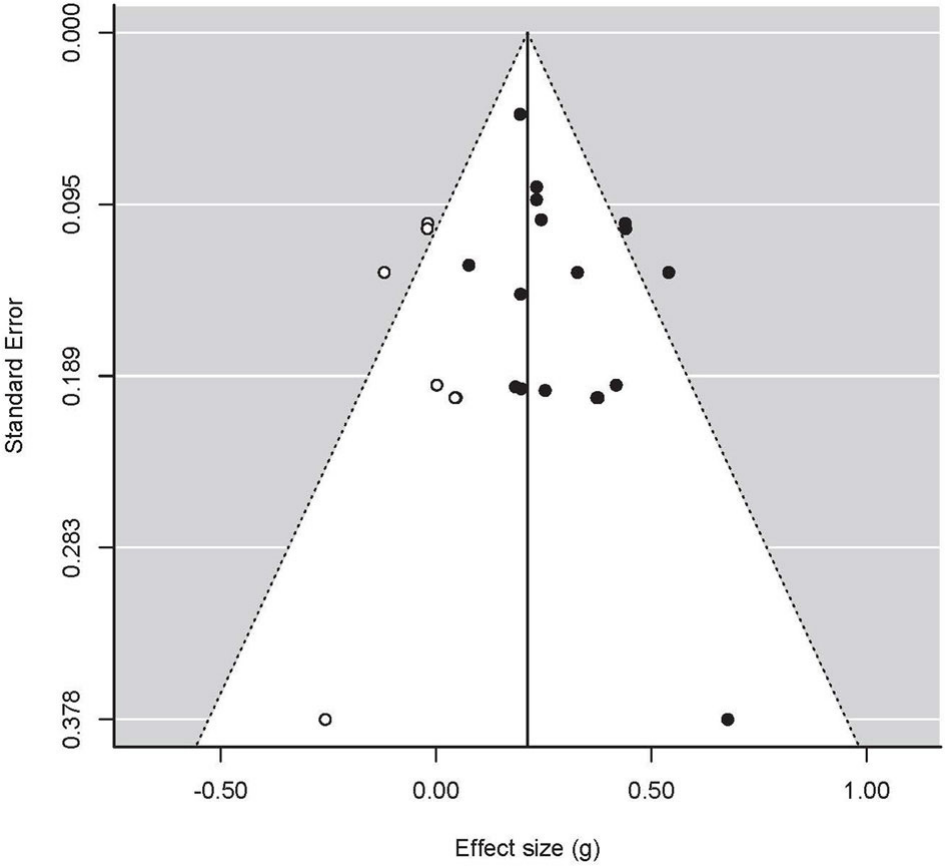
**Funnel plot for studies concerning Positive Affect, with treatment effects on the x-axis and the standard error on the y-axis**. Closed circles are original data, open circles represent filled-in data based on the trim-and-fill method. Dotted lines represent 95% confidence interval around the mean.

**Figure 2:**
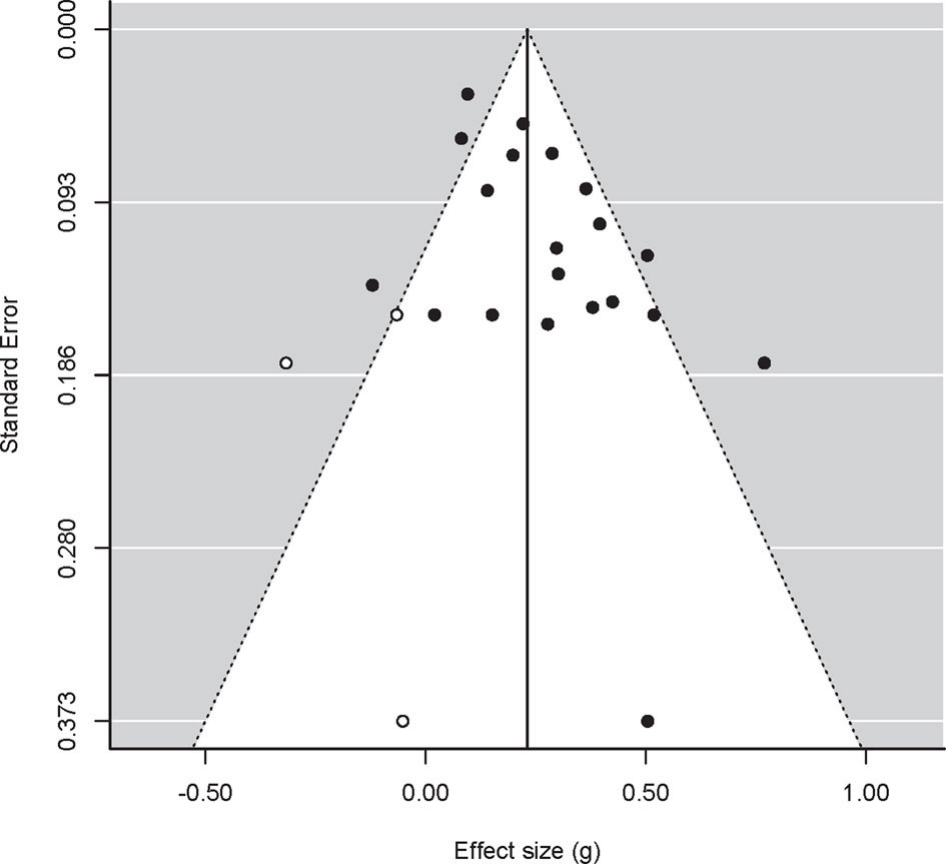
**Funnel plot for studies concerning Negative Affect, with treatment effects on the x-axis and the standard error on the y-axis**. Closed circles are original data, open circles represent filled-in data based on the trim-and-fill method. Dotted lines represent 95% confidence interval around the mean.

**Figure 3:**
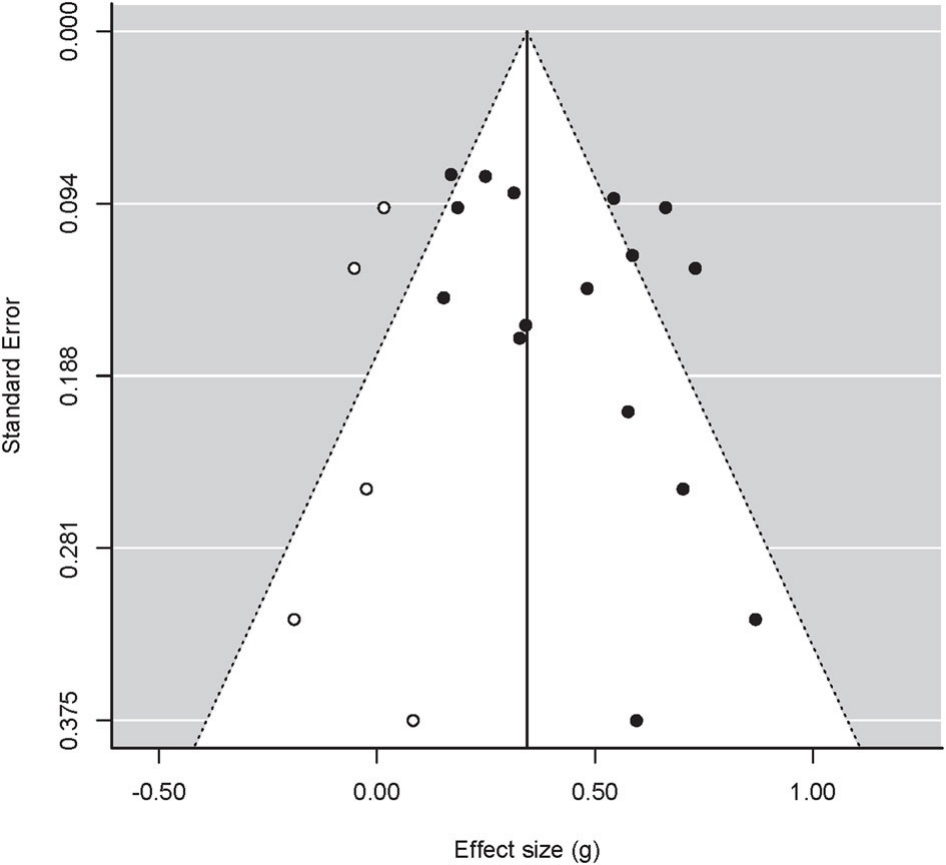
**Funnel plot for studies concerning both affects combined, with treatment effects on the x-axis and the standard error on the y-axis**. Closed circles are original data, open circles represent filled-in data based on the trim-and-fill method. Dotted lines represent 95% confidence interval around the mean.

So far, instruction seems to be a crucial factor. When combining effect sizes from the three affect levels, task-relevant instructions (i.e., explicit and explicit-converted) showed a medium effect size (*g* = 0.3182) and task-irrelevant instructions (i.e., implicit) had a negligible effect size (*g* = 0.0644). To investigate the possibility of the former being caused by a publication bias, we prepared funnel plots for the three affect analyses, excluding the implicit-instruction effects and the previously identified outliers (see Figures 1–3). Visual inspection already suggests that the plots are asymmetrical with more studies with a large effect and a large standard error to the right of the mean than the left of the mean. To test for a publication bias, we followed the approach used by Bakker et al. (2012; cf. Francis, 2012), which involves the use of Egger's regression test and Ioannidis and Trikalinos (2007) test of an excess of significant outcomes. In addition, we applied the trim and fill method (Duval and Tweedie, 2000) to correct for potential funnel plot asymmetry due to publication bias.

The funnel plot of positive-affect studies (*k* = 17) is given in Figure 1 and appears to be asymmetric. Indeed, Egger's regression test was significant at α = 0.10 (which is the commonly used nominal significance level for these analyses; Bakker et al., 2012; Francis, 2012): *Z* = 1.87, *p* = 0.062. The use of trim and fill suggested seven missing studies on the left-hand side of the funnel plot, which lowered the estimated effect to 0.212 (95% CI: 0.136, 0.288). Power computations on the basis of the estimated effect size (i.e., *g* = 0.282) showed that the average power of the 17 studies was 0.54. On the basis of this power calculation, one would expect 9.2 significant outcomes. Given that nine of studies showed a significant outcome, there does not appear to be an excess of significant outcomes in this set of studies.

In the analysis of negative affect (*k* = 20; see Figure 2), Egger's regression test also indicated funnel plot asymmetry: *Z* = 2.22, *p* = 0.027. The use of trim and fill suggested three missing studies, which lowered the estimated effect from 0.260 to 0.231 (95% CI: 0.148, 0.314). Power computations on the basis of the estimated effect size (i.e., 0.260) provided a mean power over studies of 0.61. On the basis of that power calculation the expected number of significant outcomes was 12.2. Given that 13 of the studies concerning negative affect showed a significant outcome, there is no clear excess of significant outcomes in this set of studies.

Figure 3 depicts the funnel plot with effect sizes related to both affects (*k* = 16). Egger's regression test again highlighted an asymmetric funnel plot: *Z* = 1.73, *p* = 0.083. Trim and fill suggested five missing studies on the left-hand side of the funnel, which led to an estimated effect of 0.344 (95% CI: 0.230, 0.458). In this subset, the power analyses on the basis of the uncorrected mean effect size (*g* = 0.425) showed that the power averaged 0.78. On this basis, 12.4 significant outcomes are to be expected, which compares well to the dozen significant outcomes in this subset of studies. Hence, there does not appear to be an excess of significant outcomes in this analysis.

Taken together, there are some indications of publication bias in all three sets of effects. The trim and fill corrections led to lower estimate effect sizes, which all remained significant and equaled 0.21 for positive effect, 0.23 for negative affect, and 0.34 for both affects.

## Discussion

The meta-analysis revealed a reliable overall effect of affective stimuli on approach-avoidance tendencies in the studies collected for the meta-analysis, which ranged from 1999 to 2012. Overall, we found significant small-to-medium sized effects for all three valence conditions. It can be concluded that positive and negative affect have similarly sized effects on the approach-avoidance task. This implies that appetitive and aversive behaviors can be evoked by positive and negative stimuli with equal strength, and that not only one type of affect or one type of action tendency is responsible for the compatibility effect. The evidence for a link between action tendencies and affect, however, was clearly moderated by a number of variables. Due to the low number of studies in some cells, the absence of significance of course has to be treated with caution, and does not necessarily imply an absence of effect. Nevertheless, some consistent patterns of results seem to emerge from the meta-analysis.

By far the most important moderator of the compatibility effect was instruction. A consistent finding across all analyses was a non-significant overall effect when instructions did not require conscious appraisals of the affective valence of stimuli. The relation between affect and approach and avoidance is implicit in these task-irrelevant studies, because participants were instructed to evaluate a feature of the presented stimulus other than its affective valence. This absence of effect seems especially true for tasks involving actual arm movements and is in line with the conclusions of Rotteveel and Phaf (2004). Despite the vertical button stand potentially being less liable to automatic associations between affect and approach-avoidance movements than the other tasks requiring movements in the sagittal plane (cf. Alexopoulos and Ric, 2007), the implicit studies with the joystick/lever still yielded similarly small effect sizes. In general, there seems to be little evidence for a direct or automatic link between affective information processing and arm flexion and extension, irrespective of whether the movements are made in the horizontal or the vertical direction.

The task-irrelevant instructions in the feedback-joystick task may present an exception to the absence of effect in implicit conditions. This does not necessarily point to an automatic link, but may depend on the interpretation that is offered to the participants by the zooming feature. Najmi et al. (2010), for instance, assessed approach-avoidance tendencies in individuals with contamination-related obsessive-compulsive symptoms with the feedback-joystick task. They obtained rather large effects, although participants were instructed to respond to the irrelevant orientation of stimuli. As discussed earlier, the zooming feature has been shown to be resistant to cognitive re-interpretations. Rinck and Becker (2007) in their second experiment, which was not included in the current meta-analysis, phrased the instructions so that pulling the joystick was described as pulling it away from the stimulus (i.e., avoidance), and pushing the joystick as pushing it toward the stimulus (i.e., approach). This is also referred to as an object-related frame of reference, which contrasts to a self-related frame of reference (i.e., pulling the joystick toward the self vs. pushing it away from the self). In this study, however, pulling the joystick still increased, whereas pushing the joystick decreased the size of the stimulus. Consequently, the feedback was able to override the object-related instructions and they still obtained a self-related compatibility effect. Pulling away from positive stimuli was faster than pushing toward positive stimuli and pushing toward negative stimuli was faster than pulling away from negative stimuli. The object-related frame of reference in this experiment, moreover, did not result in a smaller effect relative to the self-related frame of reference in their third experiment. Thus, instead of the arm movements, the interpretation provided by the zooming function most likely drives the compatibility effect observed with the feedback-joystick task.

With respect to the irrelevant instructions, the results from the present meta-analysis correspond to those of the experimental study of Krieglmeyer and Deutsch (2010). They directly compared different measures of approach-avoidance behavior. When participants were instructed to respond to the grammatical category of emotional words (i.e., task-irrelevant instructions), the manikin task and the feedback-joystick task but not the joystick task were sensitive to valence. In the manikin task, an abstract manikin on the screen is controlled by simple button presses. Only the distance of the manikin to the stimulus varies but not the distance between stimulus and self. This task only involves key presses but no arm flexion or extension. The manikin task may also prime the participants with a particular interpretation of the movements on the screen, and therefore may be less indicative of an automatic link of affect with approach and avoidance behaviors.

A further argument against an automatic link was that the effect was not clearly moderated by type of stimulus. All affective stimuli yielded a significant effect on approach-avoidance behaviors, but there was no significant difference between them. At first sight this seems at odds with the idea of emotional facial expressions being evolutionary prepared stimuli (Öhman, 1986), and thus receiving processing priority. If anything, there was even a tendency for facial expressions to be less effective in initiating approach-avoidance behavior than all other stimuli. In their third experiment, Rotteveel and Phaf (2004) presented facial expressions as primes (100 ms) prior to mildly affective scenes (150 ms), which participants should evaluate by flexing or extending the arm. If there is an automatic link to action-tendencies, the prime faces should influence arm flexion and extension more so than the targets. Importantly, they only found an effect on arm flexion and extension in the responses to the mildly affective scenes. In sum, affective processing of the faces might occur more automatically than of mildly affective scenes, but the evolutionary preparation does not result in privileged processing in the approach-avoidance task.

The absence of a differential effect of type of task also suggests that there is no fixed link between flexion and extension movement (i.e., biceps and triceps muscle activation) and approach and avoidance. Our results revealed a reliable effect for all tasks and there was no indication that the effect differed between tasks. The three-button stand vertical stand and the horizontal joystick/lever appear to measure similar conceptual mechanisms. Compatibility effects, therefore, cannot be explained in terms of a horizontal distance-regulation account.

A diametrically opposed frame of reference did not clearly affect the effect size with the joystick/lever task. Instructions that induced a self-related frame of reference were called explicit instructions in the meta-analysis. With explicit instructions, participants are encouraged to interpret a pulling movement as approach (i.e., move the joystick toward the self) and a pushing movement as avoidance (i.e., move the joystick away from the self). We found the effect of explicit instructions not to be significantly different from what we called explicit-converted instructions, and, in fact, we even found a trend for explicit-converted instructions to yield larger effects than explicit instructions. Explicit-converted instructions mostly reverse the coupling between valence and the flexor and extensor movements. This is usually achieved by inducing an object-related frame of reference, as was done, for instance, in the study by Lavender and Hommel (2007; see also Saraiva et al., 2013). Flexing the arm now corresponds to avoidance, whereas extending the arm reflects an approach movement. There was also no strong indication in the meta-analysis for some remaining muscle specificity only with negative affect, as suggested by Saraiva et al. (2013). Because the pattern obtained by Saraiva et al. was opposite to the one postulated by Chen and Bargh (1999), this would have been revealed by larger effect sizes at the explicit-converted level relative to the explicit level with negative affect than with positive affect. This difference was indeed larger, but did not reach statistical significance. The similarity in effect sizes with explicit and explicit-converted instructions implies that there is no default association between flexion/extension and approach/avoidance, at least in the joystick/lever task.

Eder and Rothermund (2008) even obtained compatibility effects when joystick movements to the right and left were referred to as toward and away movements, respectively. Thus, their evaluative-response-coding account may be better suited to integrate these compatibility effects. According to this view, approach and avoidance behaviors are not regulated by distinct motivational mechanisms. Evaluative codes are assigned to arm movements and compatibility effects are due to a semantic match between these codes and the valence of stimuli. This account may even be extended to the button stand, because Eder and Rothermund (2008) also found compatibility effects when movements were labeled as upwards and downwards. Experiments with the button stand, however, were mostly conducted in the Netherlands. The terms used in the Dutch language (i.e., “bovenste en onderste knop”: “upper and lower button”) to phrase the instructions are not as clearly positively or negatively connoted as their English counterparts.

Eder et al. (2010) accounted for the indirect link by hypothesizing that the direction of the compatibility effect depends on how participants construe the flexor and extensor movements, which may depend on their intentions on how to deal with the affective stimuli (i.e., implementation intentions). With task-relevant instructions the intentions may have been formed as a consequence of the instructions. Such intentions do not always involve conscious planning but may be set up by unobtrusive manipulations or even inadvertently by specific features of the design (cf. Eder, 2011). In this view, the processing of affect can still be automatic but the implementation intentions forge a temporary association between the stimuli and specific responses. In the studies using implicit instructions, no intentions may have been set up. In the study by Phaf and Rotteveel (2009), however, which indeed was identified as an outlier in the meta-analysis, these intentions could have been formed by the context of other explicit-evaluation experiments that were conducted along with the arrow experiment. The large effect size in this study may be explained by the affective monitoring framework (Phaf and Rotteveel, 2012), which argues that match-mismatch processing may even have larger effects than processing intrinsically affective stimuli. According to the framework, in the Phaf and Rotteveel (2009) study the match between arrow direction and habitual eye movements made in the reading direction would have elicited strong positive affect. The large effects in the two other outlier experiments of Markman and Brendl (2005) may be explained by an additional mechanism being at work on top of the compatibility effect. Van Dantzig et al. (2009) suggested in their polarity account that the semantic correspondence between stimulus valence and the “toward” and “away” instruction labels drove the reaction time difference, irrespective of the actual movement being made and irrespective of a self or object reference frame.

There were indications of a moderate publication bias when only task-relevant instruction effects were included. All three affect analyses showed evidence of funnel plot asymmetry, but did not reveal an excess of significant findings. We would argue that this publication bias does not invalidate the whole research field (cf. Francis, 2012), but can be corrected for by the trim-and-fill method to yield plausible estimates of effect size. After correction for publication bias, still a small-to-medium effect size ranging from 0.212 to 0.344 remained. To achieve funnel plot symmetry, 15 negative or near-zero effect sizes had to be added to the 53 effects included. The moderate publication bias shows that the field has been subject to some selective publication of significant results and suppression of non-significant results. The meta-analysis also shows that the effect with task-relevant instructions is relatively robust, and can resist this publication bias. It should be noted that the publication bias would probably have been considerably larger, if we had included effect sizes calculated from the test statistics. In selective publication statistical significance thus seems to play a bigger role than the size of the effect. The common occurrence of publication bias (cf. Bakker et al., 2012) should act as a reminder to the scientific community that a single publication of a new effect cannot count as strong evidence, and certainly not as definitive “proof.” Only when the effect size and the extent of the publication bias can be judged from a meta-analysis, one can have more confidence in the finding.

In summary, our meta-analysis shows that affective evaluation can prime approach-avoidance behaviors. This effect requires affective interpretations of the movements and, conscious or non-conscious, implementation intentions with regard to the affective stimuli. The results of the meta-analysis argue against an immediate, unintentional, implicit, stimulus-based, and evolutionary based or automatized, link between affect and approach and avoidance, and against a direct link with arm flexion and extension, respectively. Instead, they support an indirect link between affect and approach-avoidance with appraisals or re-appraisals (i.e., regulation) activating specific action tendencies (cf. Frijda, 1986). In our view, affect is elicited automatically from the monitoring of processing dynamics (cf. Phaf and Rotteveel, 2012), but should be decoupled from approach and avoidance action tendencies. The anger example shows that affect and action tendencies are (nearly) orthogonal dimensions, with angry faces being unambiguously negative (i.e., reflecting obstructed processing), but being able to raise both avoidance and approach tendencies (Rotteveel and Phaf, 2004; Carver and Harmon-Jones, 2009; Krieglmeyer and Deutsch, 2013). Even smiles may foster avoidance reactions, when shown by “outgroup” members (Paulus and Wentura, 2013). Future research with the approach-avoidance task should carefully consider the interpretation of the movements suggested to the participants either by the instructions or the experimental context. If this is done, it may serve as a very useful indirect measure of such affective interpretations and intentions.

### Conflict of interest statement

The authors declare that the research was conducted in the absence of any commercial or financial relationships that could be construed as a potential conflict of interest.
