# Supplementary material for: Approach, avoidance, and affect: a meta-analysis of approach-avoidance tendencies in manual reaction time tasks
Source: Front Psychol. 2014 May 8;5:378. doi: 10.3389/fpsyg.2014.00378 (PMC4021119; doi:10.3389/fpsyg.2014.00378)
Supplement: Supplementary file 1 [file DataSheet1.PDF]

Supplementary material

Supplementary Table 1. Reviewed Studies on Positive Affect

Study	*N*	Task	Stimulus	Instruct	Valence	Design	*g*	*se*
Bakvis, Spinhoven, Zitman, & Roelofs (2011; A)	20	1	1	1	0	0	0.197	0.196
Enter, Colzato, & Roelofs (2012; A)	97	3	1	3	0	0	-0.076	0.029
Heuer, Rinck, & Becker (2007; A)	43	3	1	3	0	0	-0.035	0.135
Lange, Keijsers, Becker, & Rinck (2008; A)	30	3	1	3	0	0	0.201	0.207
Lavender & Hommel (2007; A)	28	2	3	2	0	1	0.677	0.378
Markman & Brendl (2005; A)	45	2	2	2	0	1	1.29	0.23
Markman & Brendl (2005; B)	45	2	2	1	0	1	1.061	0.223
Marsh, Ambady, & Kleck (2005; A)	48	2	3	1	0	0	0.076	0.128
Marsh, Ambady, & Kleck (2005; B)	46	2	3	1	0	0	0.196	0.144
Önal-Hartmann, Pauli, Ocklenburg, & Güntürkün (2012; A)	30	2	3	1	0	0	0.233	0.092
Phaf & Rotteveel (2009; Experiment 2A)	61	1	3	1	1	0	0.808	0.099
Roelofs, Elzinga, & Rotteveel (2005; A)	20	1	1	1	0	0	0.373	0.201
Roelofs, Minelli, Mars, van Peer, & Toni (2009; A)	20	2	1	1	0	0	0.253	0.197
Roelofs, Minelli, Mars, van Peer, & Toni (2009; B)	20	2	1	3	0	0	0.143	0.195
Roelofs, van Peer, Berretty, de Jong, Spinhoven, & Elzinga (2009; A)	22	1	1	1	0	0	0.418	0.194
Rotteveel & Phaf (2004; Experiment 1A)	48	1	1	1	0	0	0.328	0.132
Rotteveel & Phaf (2004; Experiment 2A)	48	1	1	3	0	0	0.068	0.128
Seidel, Habel, Finkelmeyer, Schneider, Gur, & Derntl (2010; A)	24	2	1	3	0	0	0.106	0.06

Table 1. *(continued)*

Study	*N*	Task	Stimulus	Instruct	Valence	Design	*g*	*se*
Seidel, Habel, Kirschner, Gur, & Derntl (2010; A)	99	2	1	1	0	0	0.244	0.103
Van Dantzig, Pecher, & Zwaan (2008; A)	56	3	2	1	0	0	0.233	0.085
Van Peer, Roelofs, Rotteveel, van Dijk et al. (2007; A)	20	1	1	1	0	0	0.376	0.201
Volman, Toni, Verhagen, & Roelofs (2011; A)	20	2	1	1	0	0	0.184	0.195
Volman, Toni, Verhagen, & Roelofs (2011; B)	20	2	1	3	0	0	0.11	0.194
Zhang, Proctor, & Wegener (2012; Experiment 1A)	32	2	2	2	0	0	0.439	0.105
Zhang, Proctor, & Wegener (2012; Experiment 1B)	32	2	2	1	0	0	0.54	0.132
Zhang, Proctor, & Wegener (2012; Experiment 1C)	32	2	2	2	0	0	0.195	0.045
Zhang, Proctor, & Wegener (2012; Experiment 1D)	32	2	2	1	0	0	0.44	0.108

Note. *N* = sample size. Task: 1 = vertical button stand, 2 = joystick/lever, 3 = feedback-joystick. Stimulus = stimulus type: 1 = emotional facial expressions, 2 = emotional words, 3 = emotional pictures. Instruct = Instruction: 1 = explicit (task-relevant), 2 = explicit-converted (task-relevant), 3 = implicit (task-irrelevant). Valence: 0 = explicitly valenced stimuli, 1 = implicitly valenced stimuli. Design: 0 = repeated measures design, 1 = independent groups design. *g* = Hedges' *g*. *se* = Standard error.

Supplementary Table 2. Reviewed Studies on Negative Affect

Study	*N*	Task	Stimulus	Instruct	Valence	Design	*g*	*se*
Bakvis, Spinhoven, Zitman, & Roelofs (2011; B)	20	1	1	1	0	0	-0.121	0.138
Enter, Colzato, & Roelofs (2012; B)	97	3	1	3	0	0	0.339	0.134
Heuer, Rinck, & Becker (2007; B)	43	3	1	3	0	0	0.115	0.107
Lange, Keijsers, Becker, & Rinck (2008; B)	30	3	1	3	0	0	0.015	0.07
Lavender & Hommel (2007; B)	28	2	3	2	0	1	0.504	0.373
Markman & Brendl (2005; C)	45	2	2	2	0	1	1.761	0.245
Markman & Brendl (2005; D)	45	2	2	1	0	1	1.847	0.25
Marsh, Ambady, & Kleck (2005; C)	48	2	1	1	0	0	0.301	0.132
Marsh, Ambady, & Kleck (2005; D)	48	2	3	1	0	0	0.395	0.105
Marsh, Ambady, & Kleck (2005; E)	46	2	3	1	0	0	0.081	0.059
Najmi, Kuckertz, & Amir (2010)	20	3	4	3	0	0	0.806	0.178
Önal-Hartmann, Pauli, Ocklenburg, & Güntürkün (2012; B)	30	2	3	1	0	0	0.297	0.118
Phaf & Rotteveel (2009; Experiment 2B)	61	1	3	1	1	0	0.518	0.154
Reinecke, Soltau, Hoyer, Becker, & Rinck (2012)	14	3	4	3	0	0	0.138	0.091
Rinck & Becker (2007; Study 1)	25	3	4	1	0	0	0.769	0.18
Rinck & Becker (2007; Study 3)	21	3	4	3	0	0	-0.106	0.243
Roelofs, Elzinga, & Rotteveel (2005; B)	20	1	1	1	0	1	0.379	0.15
Roelofs, Minelli, Mars, van Peer, & Toni (2009; C)	20	2	1	1	0	0	0.277	0.159
Roelofs, Minelli, Mars, van Peer, & Toni (2009; D)	20	2	1	3	0	0	0.098	0.156
Roelofs, van Peer, Berretty, de Jong, Spinhoven, & Elzinga (2009; B)	22	1	1	1	0	0	0.02	0.154
Rotteveel & Phaf (2004; Experiment 1B)	48	1	1	1	0	0	0.424	0.147
Rotteveel & Phaf (2004; Experiment 2B)	48	1	1	3	0	0	-0.129	0.106
Seidel, Habel, Finkelmeyer, Schneider, Gur, & Derntl (2010; B)	24	2	1	3	0	0	-0.008	0.102

Table 2. (*continued)*

Study	*N*	Task	Stimulus	Instruct	Valence	Design	*g*	*se*
Seidel, Habel, Kirschner, Gur, & Derntl (2010; B)	100	2	1	1	0	0	0.198	0.068
Van Dantzig, Pecher, & Zwaan (2008; B)	56	3	2	1	0	0	0.095	0.035
Van Peer, Roelofs, Rotteveel, van Dijk et al. (2007; B)	20	1	1	1	0	0	0.140	0.087
Volman, Toni, Verhagen, & Roelofs (2011; C)	20	2	1	1	0	0	0.151	0.154
Volman, Toni, Verhagen, & Roelofs (2011; D)	20	2	1	3	0	0	0.076	0.153
Zhang, Proctor, & Wegener (2012; Experiment 1E)	32	2	2	2	0	0	0.287	0.067
Zhang, Proctor, & Wegener (2012; Experiment 1F)	32	2	2	1	0	0	0.221	0.051
Zhang, Proctor, & Wegener (2012; Experiment 1G)	32	2	2	2	0	0	0.503	0.122
Zhang, Proctor, & Wegener (2012; Experiment 1H)	32	2	2	1	0	0	0.364	0.086

Note. *N* = sample size. Task: 1 = vertical button stand, 2 = joystick/lever, 3 = feedback-joystick. Stimulus = stimulus type: 1 = emotional facial expressions, 2 = emotional words, 3 = emotional pictures, 4 = personally relevant stimuli. Instruct = Instruction: 1 = explicit (task-relevant), 2 = explicit-converted (task-relevant), 3 = implicit (task-irrelevant). Valence: 0 = explicitly valenced stimuli, 1 = implicitly valenced stimuli. Design: 0 = repeated measures design, 1 = independent groups design. *g* = Hedges' *g*. *se* = Standard error.

Supplementary Table 3. Reviewed Studies on Both Affects

Study	*N*	Task	Stimulus	Instruct	Valence	Design	*g*	*se*
Chen & Bargh (1999; Experiment 1)	42	2	2	1	0	1	0.868	0.32
De Houwer, Crombez, Baeyens, & Hermans (2008; Experiment 4)	35	4	2	3	0	0	0.096	0.039
Eder & Rothermund (2008; Experiment 1A)	14	2	2	1	0	0	0.702	0.249
Eder & Rothermund (2008; Experiment 1B)	14	2	2	2	0	0	0.576	0.207
Eder & Rothermund (2008; Experiment 2A)	16	2	2	2	0	0	0.586	0.122
Eder & Rothermund (2008; Experiment 2B)	16	2	2	1	0	0	0.327	0.167
Eder & Rothermund (2008; Experiment 3A)	17	2	2	2	0	0	0.341	0.16
Eder & Rothermund (2008; Experiment 3B)	17	2	2	2	0	0	0.482	0.14
Eder & Rothermund (2008; Experiment 4A)	64	2	2	1	0	0	0.314	0.088
Eder & Rothermund (2008; Experiment 4B)	64	2	2	2	0	0	0.543	0.091
Eder, Rothermund, & Proctor (2010)	32	2	3	3	0	0	0.07	0.044
Jones, Young, & Claypool (2011; Experiment 1)	80	2	3	2	1	0	0.17	0.078
Krieglmeyer & Deutsch (2010; Experiment 1A)	38	4	2	1	0	0	0.73	0.129
Krieglmeyer & Deutsch (2010; Experiment 1B)	38	2	2	1	0	0	0.185	0.096
Krieglmeyer & Deutsch (2010; Experiment 2A)	48	4	2	3	0	0	0.13	0.042
Krieglmeyer & Deutsch (2010; Experiment 2B)	48	2	2	3	0	0	0.002	0.042
Krieglmeyer & Deutsch (2010; Experiment 2C)	48	3	2	3	0	0	0.094	0.043
Lavender & Hommel (2007; C)	28	2	3	2	0	1	0.595	0.375
Phaf & Rotteveel (2009; Experiment 2C)	61	1	3	1	1	0	0.662	0.096
Seidel, Habel, Finkelmeyer, Schneider, Gur, & Derntl (2010; C)	24	2	1	3	0	0	0.047	0.041
Seidel, Habel, Kirschner, Gur, & Derntl (2010; C)	100	2	1	1	0	0	0.249	0.079
Volman, Roelofs, Koch, Verhagen, & Toni (2011)	24	2	1	1	0	0	0.153	0.145

Note. N = sample size. Task: 1 = vertical button stand, 2 = joystick/lever, 3 = feedback-joystick, 4 = abstract manikin task. Stimulus = stimulus type: 1 = emotional facial expressions, 2 = emotional words, 3 = emotional pictures. Instruct = Instruction: 1 = explicit (task-relevant), 2 = explicit-converted (task-relevant), 3 = implicit (task-irrelevant). Valence: 0 = explicitly valenced stimuli, 1 = implicitly valenced stimuli. Design: 0 = repeated measures design, 1 = independent groups design. g = Hedges' g. se = Standard error.
